# The Effect of National Cancer Screening on Disparity Reduction in Cancer Stage at Diagnosis by Income Level

**DOI:** 10.1371/journal.pone.0136036

**Published:** 2015-08-18

**Authors:** Hye-Min Jung, Jin-Seok Lee, David R. Lairson, Yoon Kim

**Affiliations:** 1 Department of Health Policy and Management, Seoul National University College of Medicine, Seoul, Korea; 2 Institute of Health Policy and Management, Medical Research Center, Seoul National University, Seoul, Korea; 3 School of Public Health, University of Texas Health Science Center at Houston, Houston, Texas, United States of America; Van Andel Institute, UNITED STATES

## Abstract

**Background:**

Early detection of cancer is an effective and efficient cancer management strategy. In South Korea, the National Health Insurance administers the National Cancer Screening Program to its beneficiaries. We examined the impact of the National Cancer Screening Program on socioeconomic disparities in cancer stage at diagnosis.

**Methods:**

Cancer patients registered in the Korean Central Cancer Registry from January 1, 2010 to December 31, 2010 with a diagnosis of gastric cancer (n = 22,470), colon cancer (n = 16,323), breast cancer (n = 10,076), or uterine cervical cancer (n = 2,447) were included. Income level was divided into three groups according to their monthly contribution of National Health Insurance. We employed absolute (age-standardized prevalence rate, slope index of inequality) and relative (relative index of inequality) measures to separately examine social disparities among participants and non-participants of the National Cancer Screening Program in terms of the early-stage rate.

**Results:**

Age-standardized prevalence rates of early-stage by income group were always higher in participants than in non-participants. Furthermore, the age-standardized prevalence rate of early-stage in the low income group of the participants was also higher than that of the high income group of the non-participants. The sizes of disparities (both slope index of inequality and relative index of inequality) are smaller in participants compared to non-participants.

**Conclusion:**

National Cancer Screening Program participation reduced income disparity in cancer stage at diagnosis. Population-based cancer screening programs can be used as an effective measure to reduce income disparity in cancer care.

## Introduction

Stage at diagnosis is one of the most important prognostic factors in cancer disease identification. The World Health Organization (WHO) asserts that early detection of cancer is an effective and efficient cancer management strategy, in conjunction with prevention, evidence-based treatment, and palliative care [1]. To detect cancer at an early stage, one should consult with a doctor when symptoms are first recognized, or undergo regular screening before symptoms appear. However, because symptoms are generally absent or nonspecific at an early stage, cancer screening program is considered better than consultation after symptoms have presented.

There are two kinds of approach to cancer screening delivery. One is population-based screening, whereby eligible individuals are invited to participate in organized screening programs managed and implemented by the government or public sector. The other is opportunistic screening, which happens when an individual seeks screening or is referred by a doctor or health care professional [2]. Since population-based screening is believed to be more effective than opportunistic screening [3], many countries have organized population-based screening programs, especially for common types of cancer.

In South Korea, the National Health Insurance (NHI) administers the National Cancer Screening Program (NCSP) for NHI beneficiaries (Table 1). The program first began in 1990 and was available only to public officials or faculty of private schools, but it has gradually expanded its target population. Now, all eligible persons are included in the target group. For individuals with lower income level, the NCSP is provided without copayment, whereas others contribute 10% of the total cost as copayment.

**Table 1:** National Cancer Screening Program of South Korea.

	Target population	Detection methods	Interval (year)	Participation rate (2010)
Stomach cancer	Individuals age over 40	Upper gastrointestinography	2	44.7%
		Gastroscopy		
		Biopsy		
Colon cancer	Individuals age over 50	Fecal occult blood test	1	34.9%
		Colon study		
		Colonoscopy		
		Biopsy		
Liver cancer	High risk group^a^ age over 40	Ultrasonography	1	46.1%
		Serum alpha-fetoprotein		
Breast cancer	Woman age over 40	Mammography	2	51.4%
Cervical cancer	Woman age over 30	Pap-smear	2	40.3%

^a^ High risk group of Liver cancer includes patients with cirrhosis, chronic liver disease, hepatitis B surface antigen (HBs Ag) positive or hepatitis C virus antibody (HCV Ab) positive. However, individuals who have used medical services for biliary cancer (hepatocellular carcinoma, intrahepatic duct carcinoma and cholangiocarcinoma) during the previous two years are excluded.

An individual’s socioeconomic status is an important predictor of stage at diagnosis. Early stage diagnosis is more frequent in those with a high socioeconomic status compared to people on the lower end of the spectrum [4]. Lower participation rates, late participation, and delayed medical service use after screening in lower socioeconomic status individuals may account for these disparities. In South Korea, despite easy accessibility to the NCSP for those of the lower-class status, socioeconomic disparity also exist in cancer stage at diagnosis and cancer screening participation rate [5].

In this study, we examined the impact of the NCSP on socioeconomic disparity in cancer stage at diagnosis.

## Materials and Methods

### Source of data

The data set is derived from the following two sources: the Korea Central Cancer Registry (KCCR), a population-based national cancer registry; and the National Health Insurance Database (NHID). The KCCR, founded in 1980, collects and combines data from the computerized systems of hospitals, medical record review surveys, population-based regional cancer registries, and death certificates. It provides information about diagnosis in the International Classification of Diseases-10 code, along with date of diagnosis and stage at diagnosis. The completeness of the KCCR’s cancer incidence data in 2011 was 97.1% [6]. The NHID provides information about beneficiaries’ socio-demographic information, like age, sex, area of residence, and monthly contributions which reflect beneficiaries’ income and medical utilization before cancer diagnosis. Data from the KCCR and the NHID were merged for this study.

We identified cancer patients registered in the KCCR from January 1, 2010 to December 31, 2010, with a diagnosis of gastric cancer (C16x), colon cancer (C18x, C19x, C20x), breast cancer (C50x), or uterine cervical cancer (C53x). Liver cancer screening was not included because it targets high risk groups, which is not consistent with the purposes of population-based screening. We also excluded Medicaid enrollees, patients with a pre-existing cancer diagnosis, and patients whose stage of cancer was unknown.

Ethical approval was exempted by the institutional review board of the Seoul National University College of Medicine (H-1310-021-523) because we used publicly available data without personal identifiers.

### Outcome variable: stage at diagnosis

Stage at diagnosis was grouped into early and advanced. Derived from the Surveillance Epidemiology and End Result (SEER), the variable of KCCR’s stage at diagnosis is categorized into four stages: localized, regional, distant, and unknown. Early stage was defined as localized and advanced stage was defined as regional or distant, while unknown stage (4.5% of total subject) was excluded from the analysis. The outcome variable, early-stage rate was defined as the proportion of early cancer in the total cancer.

### Income level indicator

The Korean National Health Insurance covers the whole population of the country, except those in the lowest 3% of the income range, who are covered by Medicaid. The insured pay their monthly contribution based on their income. Monthly contribution of National Health Insurance is one of the most accurate proxy measures of individual's income in Korea. Income level was divided into three groups according to their monthly contribution.

### NCSP participation

NCSP participants were defined as those patients who were diagnosed with cancer within 1 year after undergoing cancer screening. Those who had not been screened or who were diagnosed with cancer more than 1 year after screening were defined as NCSP non-participants.

### Statistical analysis

Separate analyses were performed for men and women. We employed absolute and relative measures to separately examine social disparities among NCSP participants and non-participants in terms of the early-stage rate.

Age-standardized prevalence rate and slope index of inequality (SII) were used as the absolute measures, while relative index of inequality (RII) was used as the relative measure. SII is calculated from the slope of the weighted least squares regression, interpreted as the absolute difference in early-stage rates between those with the lowest and the highest incomes [7]. RII is calculated from the ratio of early-stage rates of those with the highest income compared to those with the lowest income. SII and RII both take into account the population size and the relative socioeconomic position of the groups [8]. Early-stage rates specific to income level were directly standardized to 10-year age range groups, using the age distribution of the 2010 South Korean population census. SII and RII were estimated by employing a log-binomial regression using PROC GENMOD of SAS statistical software [9].

We did not use odds ratios and associated RIIs computed by logistic regression analysis because the odds ratio is not a good approximation of the prevalence ratio when outcome prevalence is high [10].

All statistical tests were performed with SAS version 9.3 software, in Korean, and statistical significance was set at p-value ≤ .05 (two-tailed).

## Results

Table 2 presents gender, cancer, and NCSP participation-specific numbers of the study subjects, and the crude early-stage rates in percentiles by income group. Total number of cancer patients registered in the Korean Central Cancer Registry during 2010 is 22,470 in gastric cancer, 16,323 in colon cancer, 10,076 in breast cancer and 2,447 in uterine cervical cancer. With the exception of females with colon cancer, the crude early-stage rate of NCSP participants was always higher than that of non-participants. Cervical cancer showed the greatest difference (16.5%p) between participants and non-participants, while breast cancer showed the smallest difference (4.5%p).

**Table 2:** Gender, cancer and National Cancer Screening Program participation specific numbers of study subjects and crude early-stage rate (%) by income group.

		NCSP^a^ Participant		NCSP non-participant		Total	
		N (%)	Crude ESR^b^	N (%)	Crude ESR	N (%)	Crude ESR
**Men**							
**Stomach cancer**							
Income group	All	8,635 (100.0)	69.4	6,875 (100.0)	56.0	15,510 (100.0)	63.4
	I (high)	2,917 (33.8)	71.8	2,810 (40.9)	58.4	5,727 (36.9)	65.2
	II (middle)	3,103 (35.9)	68.8	2,275 (33.1)	55.7	5,378 (34.7)	63.3
	III (low)	2,615 (30.3)	67.4	1,790 (26.0)	52.5	4,405 (28.4)	61.4
**Colon cancer**							
Income group	All	3,573 (100.0)	45.3	6,597 (100.0)	38.5	10,170 (100.0)	40.8
	I (high)	1,167 (32.7)	45.7	2,631 (39.9)	39.9	3,798 (37.3)	41.7
	II (middle)	1,275 (35.7)	44.9	2,091 (31.7)	37.7	3,366 (33.1)	40.4
	III (low)	1,131 (31.7)	45.3	1,875 (28.4)	37.2	3,006 (29.6)	40.3
**Women**							
**Stomach cancer**							
Income group	All	3,877 (100.0)	68.6	3,083 (100.0)	56.0	6,960 (100.0)	63.0
	I (high)	1,363 (35.2)	67.8	1,272 (41.3)	58.4	2,635 (37.9)	63.3
	II (middle)	1,285 (33.1)	69.4	948 (30.7)	54.1	2,233 (32.1)	62.9
	III (low)	1,229 (31.7)	68.8	863 (28.0)	54.5	2,092 (30.1)	62.9
**Colon cancer**							
Income group	All	1,921 (100.0)	44.8	4,232 (100.0)	34.3	6,153 (100.0)	37.6
	I (high)	667 (34.7)	45.9	1,700 (40.2)	34.3	2,367 (38.5)	37.6
	II (middle)	633 (33.0)	43.8	1,323 (31.3)	36.4	1,956 (31.8)	38.8
	III (low)	621 (32.3)	44.6	1,209 (28.6)	32.0	1,830 (29.7)	36.3
**Breast cancer**							
Income group	All	4,996 (100.0)	62.1	5,080 (100.0)	57.6	10,076 (100.0)	59.8
	I (high)	1,734 (34.7)	64.2	2,161 (42.5)	59.9	3,895 (38.7)	61.8
	II (middle)	1,669 (33.4)	62.9	1,564 (30.8)	56.2	3,233 (32.1)	59.6
	III (low)	1,593 (31.9)	59.1	1,355 (26.7)	55.4	2,948 (29.3)	57.4
**Cervical cancer**							
Income group	All	951 (100.0)	74.3	1,496 (100.0)	57.8	2,447 (100.0)	64.2
	I (high)	244 (25.7)	70.1	450 (30.1)	58.2	694 (28.4)	62.4
	II (middle)	342 (36.0)	79.2	498 (33.3)	59.6	840 (34.3)	67.6
	III (low)	365 (38.4)	72.6	548 (36.6)	55.8	913 (37.3)	62.5

^a^NCSP, National Cancer Screening Program;

^b^ESR, Early-Stage Rate

**Fig 1:**
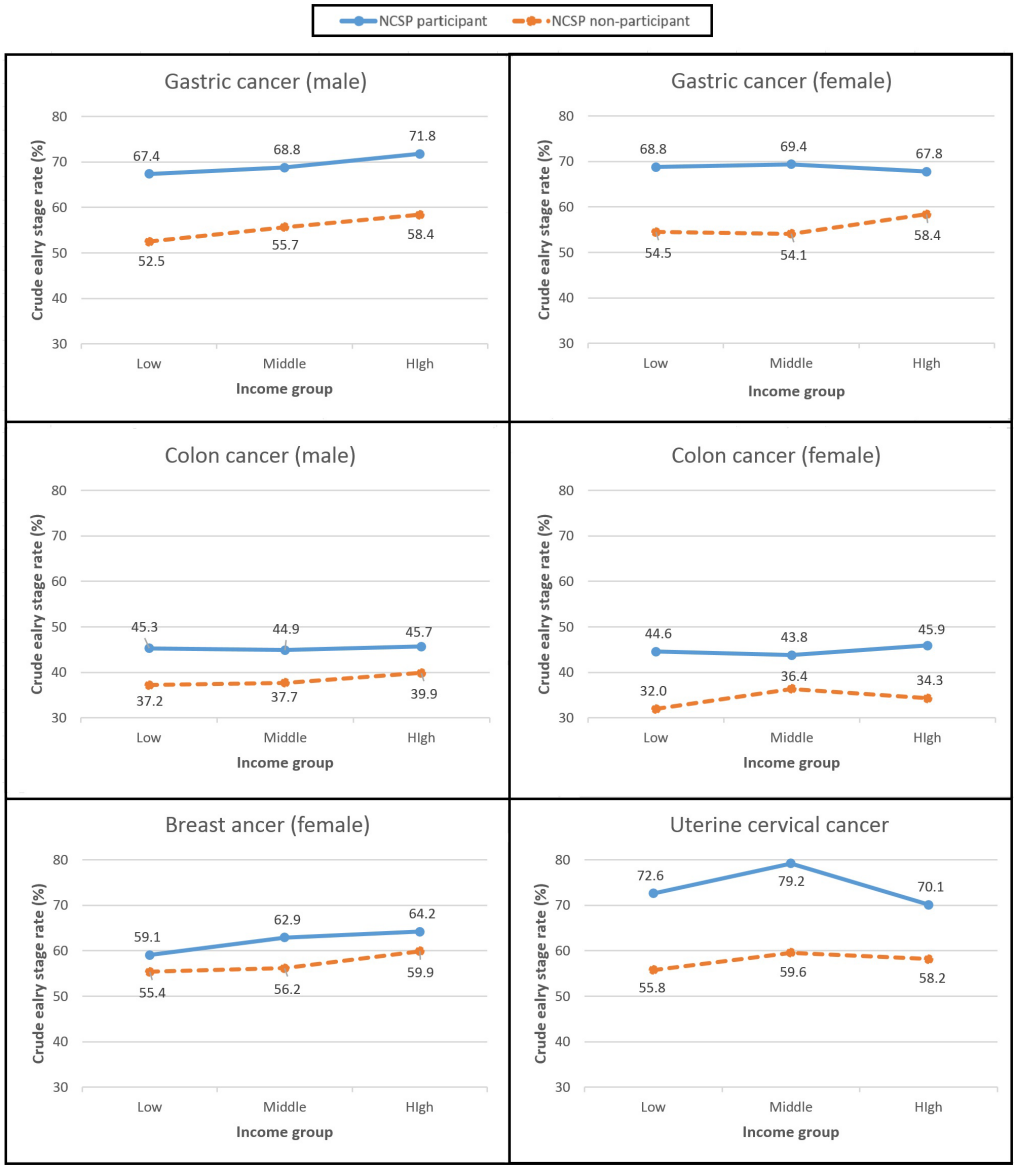
Crude early stage rate by income level according to National Cancer Screening Program participation.

As presented in Table 3, the age-standardized prevalence rates of early-stage by income group were always higher in NCSP participants than in non-participants. Furthermore, the age-standardized prevalence rate of early-stage in the low income group of the participants was also higher than that of the high income group of the non-participants. For example, in the male stomach cancer category, the age-standardized prevalence rate was 67.7 (95% C.I. 65.4–70.1) in the low income group of NCSP participants, whereas the rate was 62.4 (95% C.I. 60.2–64.6) in the high income group of NCSP non-participants. The phenomenon early stage rate (ESR) in low income group of NCSP participant was higher than in high income group of NCSP non-participant was more apparent in Fig 1.

**Table 3:** Age-standardized prevalence rates (95% CI), prevalence difference (PD) and relative index of inequality (RII) of early-stage by National Cancer Screening Program participation.

		NCSP^a^ Participant		NCSP non-participant		Total	
		Estimate	p-value	Estimate	p-value	Estimate	p-value
**Men**							
**Stomach cancer**							
Income group	I (high)	72.6 (70.5–74.7)		62.4 (60.2–64.6)		67.5 (66.0–69.0)	
	II (middle)	69.0 (67.0–71.0)		55.4 (52.8–58.0)		63.3 (61.7–64.9)	
	III (low)	67.7 (65.4–70.1)		52.0 (49.0–55.0)		61.3 (59.4–63.2)	
	SII (95% CI)	-7.1 (-10.7–3.5)	0.0001	-10.5 (-14.8–6.1)	<0.0001	-8.6 (-11.4–5.8)	<0.0001
	RII (95% CI)	0.90 (0.86–0.95)	<0.0001	0.82 (0.76–0.89)	<0.0001	0.87 (0.03–0.91)	<0.0001
**Colon cancer**							
Income group	I (high)	46.5 (43.2–49.8)		42.7 (40.5–44.8)		43.9 (42.1–45.6)	
	II (middle)	44.2 (41.2–47.1)		38.1 (35.9–40.4)		40.4 (38.6–42.2)	
	III (low)	44.8 (41.6–48.0)		37.4 (35.1–39.8)		40.1 (38.2–42.0)	
	SII (95% CI)	-1.3 (-7.3–4.7)	0.675	-5.3 (-9.6–1.0)	0.016	-3.9 (-7.5–0.4)	0.029
	RII (95% CI)	0.97 (0.85–1.11)	0.663	0.86 (0.77–0.96)	0.008	0.90 (0.83–0.98)	0.017
**Women**							
**Stomach cancer**							
Income group	I (high)	67.9 (65.1–70.7)		60.0 (56.7–63.3)		64.3 (62.2–66.4)	
	II (middle)	68.7 (66.0–71.4)		54.9 (51.3–58.5)		62.9 (60.7–65.1)	
	III (low)	68.6 (65.8–71.4)		55.1 (51.2–59.0)		63.4 (61.1–65.7)	
	SII (95% CI)	1.1 (-4.3–6.4)	0.701	-7.3 (-13.8–0.9)	0.027	-2.6 (-6.8–1.6)	0.217
	RII (95% CI)	1.02 (0.94–1.10)	0.701	0.87 (0.78–0.98)	0.024	0.96 (0.90–1.02)	0.215
**Colon cancer**							
Income group	I (high)	44.1 (40.1–48.1)		36.0 (33.4–38.7)		38.4 (36.2–40.5)	
	II (middle)	44.4 (40.4–48.4)		37.5 (34.8–40.2)		39.6 (37.4–41.9)	
	III (low)	44.1 (40.2–48.1)		33.5 (30.6–36.3)		37.4 (35.1–39.7)	
	SII (95% CI)	-2.4 (-10.6–5.8)	0.567	-3.6 (-8.9–1.7)	0.188	-3.3 (-7.7–1.2)	0.154
	RII (95% CI)	0.95 (0.79–1.14)	0.605	0.90 (0.78–1.05)	0.200	0.92 (0.82–1.04)	0.193
**Breast cancer**							
Income group	I (high)	64.4 (61.9–66.9)		59.9 (57.7–62.1)		62.0 (60.3–63.6)	
	II (middle)	63.5 (60.7–66.3)		56.8 (53.9–59.7)		60.2 (58.2–62.2)	
	III (low)	60.7 (57.7–63.6)		54.9 (51.9–57.9)		57.7 (55.6–59.8)	
	SII (95% CI)	-7.2 (-12.1–2.2)	0.005	-7.3 (-12.3–2.2)	0.005	-7.2 (-10.8–3.7)	<0.0001
	RII (95% CI)	0.89 (0.82–0.97)	0.005	0.88 (0.81–0.96)	0.005	0.89 (0.83–0.94)	<0.0001
**Cervical cancer**							
Income group	I (high)	74.8 (69.0–80.6)		62.1 (56.9–67.4)		66.3 (62.2–70.4)	
	II (middle)	81.4 (76.9–85.8)		63.4 (58.9–67.9)		70.6 (67.4–73.8)	
	III (low)	72.5 (66.9–78.1)		57.8 (53.6–62.1)		63.5 (60.2–66.9)	
	SII (95% CI)	-0.5 (-10.5–9.5)	0.917	-7.8 (-16.9–1.3)	0.094	-5.0 (-11.9–1.9)	0.156
	RII (95% CI)	0.99 (0.87–1.13)	0.848	0.88 (0.76–1.02)	0.099	0.92 (0.83–1.02)	0.125

^a^NCSP, National Cancer Screening Program.

Table 3 also shows SII and RII values. SII measures the absolute difference in early-stage rate between those with the lowest and highest incomes. For example, in the female stomach cancer category, the absolute difference of early-stage rate between those with the lowest and highest incomes is 1.1%p (p = .701) among NCSP participants. This means that the individual with the lowest income is diagnosed with early stage cancer at 1.1%p more than the individual with the highest income. On the other hand, among NCSP non-participants, the individual with the lowest income is diagnosed with early stage cancer at 7.3%p (p = .027) less than the individual with the highest income. Almost every SII column except the NCSP participant female stomach cancer shows a negative estimate value. This means that individuals in the low income group are diagnosed with early stage cancer less than individuals in the high income group are. However, the sizes of disparities are smaller in NCSP participants compared to NCSP non-participants.

RII values in Table 3 show the prevalence ratio between the two ends of the income hierarchy. The closer the RII value is to 1.00, the smaller the disparities. A negative RII value indicates that the early-stage rate of the individuals with the lowest income is lower than that of the individuals with the highest income. For example, in the male colon cancer category, the RII among NCSP participants is 0.97 (p = .663), which demonstrates that there is no significant difference in the early-stage rate between groups. However, the RII of 0.86 (p = .008) among NCSP non-participants shows that there is a significant difference in early-stage rate between individuals with the highest versus lowest income.

## Discussion

Consistent with previous studies on socioeconomic disparity in early diagnosis of cancer, this study found a trend of increasing early-stage rates as income increased. According to a study based on the Metropolitan Detroit Cancer Surveillance System, the early-stage rate of colon, lung, breast, uterine cervix, and prostate types of cancer was significantly higher in the uppermost socioeconomic position than in the bottom [4]. Another study using SEER data from the National Cancer Institute and National Longitudinal Mortality Study, also showed that the early-stage rate of breast and prostate cancer was significantly higher among those in top socioeconomic positions [11]. However, in cases of colorectal cancer, evidence demonstrated that the disparity was not significant [11,12]. Socioeconomic disparity in cancer stage distribution may be attributed to the disparity in health care access and use, particularly in relation to cancer screening [5,13].

This study suggests that NCSP participation reduced income disparities in early-stage rate. Significant income disparities of early stage diagnosis in female stomach cancer and male colon cancer categories almost disappeared after NCSP participation was taken into account. In female colon cancer and uterine cervical cancer categories, marginally significant income disparities also became non-significant after NCSP participation was taken into account. Meanwhile, significant income disparities in male stomach cancer and female breast cancer categories remained, but the size of disparities decreased. One possible explanation for the various results is that we could not obtain information about precursor lesions due to the limited variables available in the KCCR. Cancer screening aims to not only detect and cure early cancer, but also prevent cancer via detecting precursor lesions [2]. Therefore, as a result of exclusion of precursor lesion information, the impact of NCSP was likely underestimated.

In our study, the disparity gap in the colorectal cancer category was relatively small in every category (male vs. female, NCSP participant vs. non-participant). We assume that colon cancer screening is not so popular even among individuals in the high socioeconomic class, because the screening procedure causes large discomfort. Therefore, there may be small differences in access and use of colon cancer screening among all socioeconomic classes. Another finding is that disparities were definite in male, but not female, cancer patients. One potential explanation is that fewer women with high incomes actively participate in cancer screening, compared to the number of participating high income men. A husband’s health is considered more important than that of wife, because strong Confucian and patriarchal traditions remain in South Korea. It can affect the screening behavior.

Some research has shown that cancer detected via screening has a better early-stage rate than cancer detected after symptoms arise [2,12,14,15]. In our study, cancer patients who participated in NCSP always showed a higher early-stage rate than that of non-participants. That means NCSP significantly attributed to overcome socioeconomic disadvantages. Furthermore, the low income group among NCSP participants shows a higher early-stage rate than that of the high income group among NCSP non-participants.

Our research does have some limitations. First, the KCCR probably has missing cases. However, the missing cases were too few in number to induce biased results. In fact, the missing rate of KCCR data in 2011 was only 2.9% [6].

Second, we could not include opportunistic cancer screening data. In Korea, socioeconomic disparities in participation rates are decreasing in population-based screening (NCSP), whereas these disparities are widening in opportunistic screenings [16].

It is possible that early cancer patients who were classified as being in the NCSP non-participant group but underwent opportunistic cancer screening were more prevalent in the high income group. This could mean there was a overestimated disparity in the NCSP non-participant group, and subsequently an overestimation of the disparity reduction.

Third, the proxy measure 'monthly contribution' is based on individual’s income. Thus, it is possible that person with low incomes from wealthy households can distorted the result.

Finally, reduced ESR difference among income level always does not mean reduced health disparity because of the potential for length time bias which may vary by tumor location.

Despite these limitations, this is the first study analyzing the impact of population-based screening program on socioeconomic disparities in relation to cancer stage at diagnosis. Furthermore, the results are highly generalizable because the study includes all Korean cancer patients diagnosed in one year.

## Conclusions

In this study, we examined the impact of the NCSP on socioeconomic disparity in cancer stage at diagnosis. This study showed that there were disparities in cancer stage at diagnosis in Korea but the size of disparities were smaller in NCSP participants compared to non-participants. This finding suggests that population based cancer screening program can be used as an effective measure to reduce income disparity in cancer stage at diagnosis.
